# Sulfate is transported at significant rates through the symbiosome membrane and is crucial for nitrogenase biosynthesis

**DOI:** 10.1111/pce.13481

**Published:** 2019-01-28

**Authors:** Sebastian Schneider, Arno Schintlmeister, Manuel Becana, Michael Wagner, Dagmar Woebken, Stefanie Wienkoop

**Affiliations:** ^1^ Division of Molecular Systems Biology, Department of Ecogenomics and Systems Biology University of Vienna Vienna Austria; ^2^ Division of Microbial Ecology, Department of Microbiology and Ecosystem Science, Research Network “Chemistry Meets Microbiology” University of Vienna Vienna Austria; ^3^ Large‐Instrument Facility for Advanced Isotope Research University of Vienna Vienna Austria; ^4^ Estación Experimental de Aula Dei CSIC Zaragoza Spain

**Keywords:** legume nodules, nanoSIMS, nitrogen fixation, stable isotope labelling, sulfur deficiency, symbiotic sulfate transporter (SST1)

## Abstract

Legume–rhizobia symbioses play a major role in food production for an ever growing human population. In this symbiosis, dinitrogen is reduced (“fixed”) to ammonia by the rhizobial nitrogenase enzyme complex and is secreted to the plant host cells, whereas dicarboxylic acids derived from photosynthetically produced sucrose are transported into the symbiosomes and serve as respiratory substrates for the bacteroids. The symbiosome membrane contains high levels of SST1 protein, a sulfate transporter. Sulfate is an essential nutrient for all living organisms, but its importance for symbiotic nitrogen fixation and nodule metabolism has long been underestimated. Using chemical imaging, we demonstrate that the bacteroids take up 20‐fold more sulfate than the nodule host cells. Furthermore, we show that nitrogenase biosynthesis relies on high levels of imported sulfate, making sulfur as essential as carbon for the regulation and functioning of symbiotic nitrogen fixation. Our findings thus establish the importance of sulfate and its active transport for the plant–microbe interaction that is most relevant for agriculture and soil fertility.

## Abstract 2

This article provides a comprehensive explanation for the importance of the nodule‐specific sulfate transporter and the role of sulfate by dissecting the sulfur distribution across the nodule tissue and directly linking sulfate incorporation to nitrogenase biosynthesis.

## 1 INTRODUCTION

Nitrogen (N) is an essential plant nutrient and the most limiting factor for plant productivity and agricultural production worldwide. However, the symbiosis established between leguminous plants and certain soil bacteria, collectively known as rhizobia, is able to overcome N limitation by providing nutritional benefits for both partners (Lugtenberg & Kamilova, 2009). Symbiotic nitrogen fixation (SNF) is the largest natural source of N in agricultural systems (Smil, 1999). This process enables plants to trap atmospheric N_2_ to satisfy their N demand and is important for sustainable agriculture that faces the necessity of reducing the input of N‐fertilizers because of their negative eutrophication effects (Peoples, Herridge, & Ladha, 1995) and their contribution to nitrous oxide emissions from soil that accelerate global warming (Smith, Mosier, Crutzen, & Winiwarter, 2012). The legume–rhizobia interaction leads to the formation of a new specialized plant organ, the nodule, where SNF takes place. In many legumes, rhizobia colonize roots through formation of infection threads near the tip of the epidermal root hair cells and are released into cortical cells via endocytosis (Oldroyd, Murray, Poole, & Downie, 2011). The developing nodule cells continue to proliferate, and rhizobia become enclosed individually or in small groups within a new organelle, the symbiosome. This consists of the bacteroids (differentiated rhizobia that fix N_2_), a plant‐derived symbiosome membrane (SM), and a symbiosome space (SS) between the bacteroids and the membrane (Mellor, 1989). The SM is an interface for metabolic exchange between the two symbiotic partners. Essentially, this exchange includes ammonium produced by the bacteroid and photosynthetically derived organic acids produced by the plant (Udvardi & Poole, 2013).

During nodule development, both partners undergo coordinated differentiation that involves global changes in gene expression (Colebatch et al., 2002; Colebatch et al., 2004; Fedorova et al., 2002). The major rhizobial proteins induced through symbiosis and enabling SNF are the nitrogenase components (Child, 1975; Scowcroft & Gibson, 1975). The nitrogenase complex consists of an Fe‐protein (dinitrogenase reductase) and a MoFe‐protein (dinitrogenase). The Fe‐protein is a homodimer (γ_2_) containing an Fe_4_S_4_ cluster, whereas the MoFe‐protein is a heterotetramer (α_2_β_2_) comprising Fe_8_S_7_ clusters (P‐clusters) at the α‐β interface and FeMo‐cofactors (MoFe_7_S_9_C‐homocitrate) within the α subunits (Rubio & Ludden, 2008). Consequently, the synthesis of nitrogenase requires a considerable supply of sulfur (S), and an insufficiency in this element drastically affects the symbiotic interaction (DeBoer & Duke, 1982; Udvardi & Poole, 2013).

Sulfur is an indispensable and limiting nutrient for all living organisms (Zhao, Wood, & McGrath, 1999). Sulfate is actively taken up and assimilated by plants and many microorganisms via specific sulfate transporters. It is converted to the nutritionally important S‐containing amino acids cysteine (Cys) and methionine (Met), which are necessary for protein biosynthesis (Kopriva & Rennenberg, 2004; Leustek & Saito, 1999). Furthermore, S is used for the formation of coenzymes, ligands, and FeS clusters of enzymes (Davidian & Kopriva, 2010). Hence, S malnutrition in plants leads to perturbations in amino acid pools and causes reduction of biomass production and chlorophyll content (Nikiforova et al., 2005). Sulfur deficiency of soils has gained increased attention over the past three decades on a worldwide scale (Scherer, 2009). However, although this nutrient deficiency reduces crop yield and quality, its crucial effects on SNF have been overlooked. The physiology of plant sulfate transport has been extensively studied (Buchner, Takahashi, & Hawkesford, 2004; Smith, Ealing, Hawkesfordt, & Clarkson, 1995; Takahashi et al., 2000), and several genes encoding high‐affinity sulfate transporters have been isolated and characterized (Smith et al., 1997; Takahashi et al., 1997; Yoshimoto, Takahashi, Smith, Yamaya, & Saito, 2002). It was shown that plant organs have different affinities towards sulfate transport, which enables an efficient uptake throughout the whole plant (Hawkesford, 2003; Kreuzwieser, Herschbach, & Rennenberg, 1996; Takahashi et al., 2000). In early work, we provided evidence that sulfate transporters play a role in SNF (Wienkoop & Saalbach, 2003). Based on proteomic analyses of isolated SM from nodules of the model legume Lotus japonicus, the sulfate transporter SST1 was identified and shown to be specifically expressed in the nodules (Krusell et al., 2005). Subsequently, SST1 was localized to the SM (Wienkoop & Saalbach, 2003) and suggested to be responsible for the transport of sulfate from the plant to the bacteroids (Krusell et al., 2005). These authors proposed that SST1 is also able to transport molybdate, but this seems not to be the case because, very recently, molybdate‐specific transporters have been detected in Medicago truncatula nodules (Gil‐Díez et al., 2018; Tejada‐Jiménez et al., 2017).

There is increasing evidence that SST1 is imperative for nodule activity, but the role of S in SNF is poorly defined (Kalloniati et al., 2015; Krusell et al., 2005). In this study, we provide evidence for the crucial role of active transport of high levels of S through the SM and its significant accumulation in the bacteroids, which is essential for nitrogenase biosynthesis.

## 2 MATERIALS AND METHODS

### 2.1 Plant growth

Seeds of L. japonicus Gifu B‐129 and its mutant derivative *sst1‐1* (sym13) were kindly provided by Niels Sandal and Jens Stougaard (Aarhus University, Denmark). Surface‐sterilized seeds were germinated on agar plates in B&D nutrient solution (Broughton & Dilworth, 1971) solidified with 0.8% plant agar. After 7 days, seedlings were inoculated with Mesorhizobium loti strain R7A and transferred to pots containing sterilized clay granules (Seramis, Westland Horticulture, Dungannon, UK) soaked in B&D nutrient solution supplemented with 1‐mM KNO_3_. Plants were then grown in N‐free B&D solution in a climate chamber with a 16‐hr photoperiod and 22°C/18°C (day/night) regime and a relative humidity of 70% as described (Kalloniati et al., 2015; Krusell et al., 2005).

### 2.2 ^34^ S‐sulfate metabolic labelling in planta

Three weeks after infection, plants were checked for sufficient nodulation, irrigated for 2 days with water to wash out the nutrients from the substrate, and divided into two subsets: control plants and plants for ^34^S uptake. For stable isotope labelling *in planta* (^34^S SILIP), Mg^32^SO_4_^.^7H_2_O, K_2_^32^SO_4_, Mn^32^SO_4_^.^H_2_O, Zn^32^SO_4_^.^7H_2_O, Cu^32^SO_4_^.^5H_2_O, and Co^32^SO_4_^.^7H_2_O in the B&D solution were replaced by MgCl_2_, K_2_HPO_4_, MnCl_2_^.^2H_2_O, ZnCl_2_, CuCl_2_^.^2H_2_O, and CoCl_2_, respectively. This was necessary to maintain the same nutrient concentrations as in the original solution. Likewise, Na_2_^34^SO_4_ (90 at% ^34^S, 98% [CP]; Sigma‐Aldrich) was included as the only S source to maintain a concentration of 0.5‐mM sulfate in the B&D solution. Plants were watered with the ^34^S‐nutrient solution, and nodules were harvested after 96 hr of labelling and used for protein extraction. Control plants (without ^34^S labelling) were supplied with the same nutrient solution, but using unlabelled Na_2_^32^SO_4_.

### 2.3 Protein extraction and LC‐MS/MS analysis

Nodules were separated into two fractions as described (Larrainzar et al., 2007). Briefly, nodules were homogenized in Eppendorf tubes on ice with cold extraction buffer comprising 50‐mM HEPES (pH 7.5), 300‐mM sucrose, 10‐mM dithiothreitol (DTT), and 1‐mM phenylmethanesulfonyl fluoride. The homogenate was centrifuged (2,000 *g*, 10 min, 4°C), and the supernatant (plant fraction) was saved. The pellet containing the symbiosomes and bacteroids was washed, and the supernatant was pooled with the previous plant fraction. The remaining symbiosome pellet was resuspended in the same extraction buffer as before but omitting sucrose. The suspension was sonicated on ice for 5 min to release the bacteroids from the symbiosomes and solubilize bacteroidal proteins. The suspension was cleared by centrifugation (10,000 *g*, 15 min, 4°C), and the supernatant (bacteroid fraction) was saved. The pellet was extracted again, and the supernatant was pooled with the bacteroid fraction.

To each tube, 1.5‐ml ice‐cold acetone with 0.5% β‐mercaptoethanol was added, and the plant and bacterial proteins were precipitated overnight at −20°C. The pellet (20,000 *g*, 15 min, 4°C) was washed with 2‐ml ice‐cold methanol containing 0.5% β‐mercaptoethanol and was centrifuged again (20,000 *g*, 10 min, 4°C). The pellet was resuspended in urea buffer (8 M urea, 500‐mM HEPES), and protein concentration was determined by the dye‐binding assay (Bradford, 1976) using bovine serum albumin as standard.

Before digestion, proteins were reduced with 5‐mM DTT and alkylated with 10‐mM iodoacetamide. Alkylation was stopped with 10‐mM DTT, and samples were diluted with trypsin buffer (10% acetonitrile, 50‐mM ammonium bicarbonate, 2‐mM CaCl_2_, 5‐mM DTT) to a concentration of 2 M urea. Poroszyme immobilized trypsin beads (1:10 [vol:wt]; Applied Biosystems) were used for protein digestion overnight at 37°C. The digest was desalted on C_18_ stage tips (Pierce Thermo Scientific, USA).

Dried peptides were dissolved in 2% acetonitrile containing 0.1% fluoroacetic acid, and 0.5 μg of each sample was applied on a reversed phase C_18_ column (Acclaim PepMap, 3 μm, 100 Å, 75 μm × 50 cm; Thermo Fisher Scientific, Austria). Peptides were eluted from the column with a 120‐min linear gradient from solvent A (2% acetonitrile, 0.1% fluoroacetic acid) to 90% solvent B (100% acetoniltrile, 0.1% fluoroacetic acid) with a flow rate of 300 nl/min (UHPLC, Ultimate 3000; Thermo Scientific Dionex, Sunnyvale, CA, USA). Mass spectrometry (MS) measurements were performed on an LTQ‐Orbitrap Elite (Thermo Fisher Scientific; Bremen, Germany) using the following settings: full scan range 350–1800 *m*/*z*, resolution 120,000, profile mode, maximum 20 MS/MS scans (activation type CID), repeat count 1, repeat duration 30 s, exclusion list size 500, exclusion duration 60 s, charge state screening enabled with rejection of unassigned and +1 charge states, minimum signal threshold 5000.

### 2.4 Protein identification

MaxQuant (Cox & Mann, 2008) version 1.6.0.1 was used for protein identification and relative quantification. Spectra were matched against combined proteome databases of M. loti (Rhizobase, Kazusa DNA Research Institute, Japan) and L. japonicus (Lotus Base [Mun, Bachmann, Gupta, Stougaard, & Andersen, 2016], v3.0 proteins) using Andromeda (Cox et al., 2011). Oxidation at Met and acetylation at the protein N‐terminus were set as variable modification, carbamidomethylation of Cys was set as fixed modification, and a maximum of up to two missed cleavage sites was allowed. For peptide identification, a minimum of six amino acids was required and at least two peptides were needed for protein identification. The MaxQuant evidence file was utilized to obtain the respective mass‐to‐charge ratio and retention time values of target peptides for ^34^S incorporation analysis. The MaxQuant proteinGroups file was used to receive the label‐free quantification intensities (Cox et al., 2014). Detailed protein and peptide identification, scoring, and label‐free quantification information are available in Tables S1a and S1b.

### 2.5 ^34^ S incorporation analysis

For the ^34^S protein incorporation analysis, at least two different target peptides of the most abundant bacterial nitrogenase subunits (Table S2a) and plant nodulins (Table S2b), each containing either one Cys or one Met, and a robust signal for all three isotopic peaks, were selected (Table S2). The LC‐MS system software Xcalibur (version 2.2 SP1.48, Thermo Scientific) was used for manual ion intensity extraction. First, peptide ion traces were extracted. At the peak apex, the first and the third isotopic peak of a peptide, representing the “light” monoisotopic (^32^S/^12^C/^14^N) peak and the putative “heavy” ^34^S peak (+1.99 Da), were used for calculation. To display protein turnover by the ^34^S incorporation into target peptides, the relative isotope abundance (RIA; Lyon et al., 2016) was calculated as the ratio of the heavy ^34^S intensity to the sum of heavy and light ^32^S intensities and averaging RIAs over all analysed proteotypic peptides per protein (Table S2). Due to the isotopic overlap (Herbst et al., 2013), RIA values of the different peptides were transformed by subtracting each of the respective minimal RIA values over all biological replicates for both time points.

### 2.6 Sample preparation for TEM and NanoSIMS analyses

Chemical fixation of wild‐type (wt) and mutant nodules was performed as described (Krusell et al., 2005; Madsen et al., 2010). Bisected nodules were fixed in 2.5% glutaraldehyde in 0.1 M sodium cacodylate buffer (pH 7.0) at 4°C for 24 hr. Nodules were subsequently dehydrated with two successive series of 70% and 100% ethanol, 30 min each, and they were finally embedded in LR White hard grade (London Resin Company; Reading, UK).

Semithin sections (1 μm) and ultrathin sections (150 nm) were cut using a Leica EM UC7 ultramicrotome. The semithin sections were dried on glass slides and stained with 1% toluidine blue for visual inspection by optical microscopy. For correlative imaging, ultrathin sections were serially cut, and the first section of each nodule sample was transferred to a copper grid (3.05 mm, Agar Scientific). The sections were stained with gadolinium triacetate for 30 min (Nakakoshi, Nishioka, & Katayama, 2011) and lead citrate for 8 min (Reynolds, 1963) and used for transmission electron microscopy (TEM) with a Zeiss Libra 120 electron microscope (Zeiss, Germany). The subsequent sections of each nodule were deposited onto indium tin oxide‐coated glass slides (7.1 x 7.1 x 1 mm; Präzisions Glas & Optik GmbH, Iserlohn, Germany) for nanoscale secondary ion MS (NanoSIMS) analysis.

### 2.7 RNA extraction and quantitative reverse‐transcription PCR

Approximately 40 mg of nodules were used for total RNA extraction, cDNA synthesis, and gene expression analysis as described (Fukudome et al., 2016; Matamoros et al., 2015). Quantitative RT‐PCR was performed using a 7500 Real‐Time PCR System (Applied Biosystems) and the listed primers (Table S3). The transcript levels of the leghemoglobin (Lb) isoforms 1 and 2 (Lj1/2) were calculated relative to L. japonicus ubiquitin (LjUBQ) and ATP synthase (LjATPsyn), whereas rhizobial *nifH* levels were normalized to *sigA* as described in Ott et al. (2005).

### 2.8 Statistical analysis

Data processing and statistical analysis were performed using R software (RStudio Version 1.0.153). A minimum of three biological replicates were used in all analyses. The significance of ^34^S incorporation was tested applying one‐way ANOVA with post hoc Tukey HSD (honest significant difference) and Kruskal–Wallis with post hoc Wilcoxon rank sum test and *P*‐value adjustment by the FDR method (Benjamini & Hochberg, 1995).

Data are presented as box and whisker plots, generated in R using the “ggplot2” library, presenting the first and third quartile (box), the median (second quartile in the box), and the highest and lowest data point within 1.5 interquartile ranges (whiskers). Outliers are presented as black dots. Bar plots and the respective overlaid “jitter” dot plots show the distribution of all biological replicates and were created in R using the “ggpubr” package.

### 2.9 NanoSIMS analysis and data evaluation

All measurements were carried out on an NS 50 L instrument (Cameca, Gennevilliers, France). Prior to data acquisition, analysis areas were preconditioned *in situ* by rastering of a high intensity, defocused Cs^+^ ion beam in the following sequence of high and extreme low ion impact energies (HE /16 keV and EXLIE/50 eV, respectively): HE at 100‐pA beam current to a fluence of 5.0E14 ions/cm^2^; EXLIE at 400‐pA beam current to a fluence of 5.0E16 ions/cm^2^; HE at 100 pA to a fluence of 2.5E14 ions/cm^2^. Data were acquired as multilayer image stacks obtained by sequential scanning of a finely focused Cs^+^ ion beam and simultaneous detection of secondary ions and secondary electrons. High‐resolution imaging (~40 nm probe size at 0.5‐pA beam current) was accomplished through application of a lens voltage of 5.5 kV on the Einzel lens “L1” in the primary ion column and beam divergence reduction by insertion of a diaphragm with 100‐μm inner diameter (“D1#4”) into the coaxial lens stack.

The detectors of the multicollection assembly were positioned to enable parallel detection of ^12^C^−^, ^12^C^14^N^−^, ^31^P^−^, ^32^S^−^, and ^34^S^−^ secondary ions. The electrostatic lenses and deflectors inside the spectrometer were adjusted to achieve a mass resolving power of >9,500 (according to Cameca's definition) for detection of CN^−^ and S^−^ secondary ions. During acquisition, secondary ion beam drift was corrected by automatic beam centring (utilizing the ^12^C^−^ signal as reference), as well as automatic peak centring for each of the recorded secondary ion species (utilizing the ^12^C^14^N^−^ signals as reference signal for the ^31^P^−^, ^32^S^−^ and ^32^S^−^ signals, respectively). Scanning areas were in the range from 15 × 15 to 22 × 22 μm^2^ at typically 512 × 512 pixel image resolution and 10‐ms dwell time per pixel and cycle.

**Figure 1:**
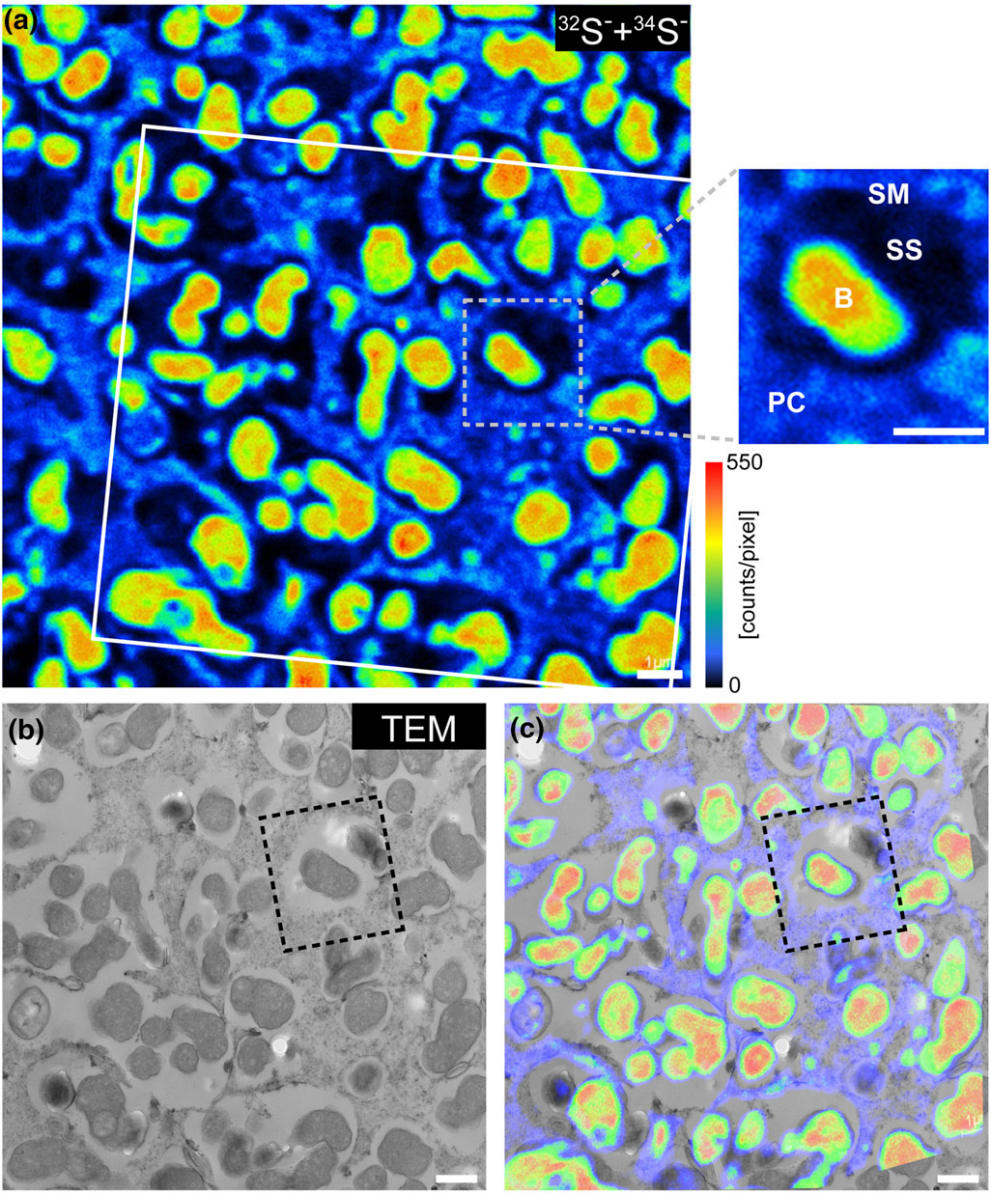
NanoSIMS high‐resolution analysis of a nodule. (a) Relative distribution of S within a thin section of a wt nodule, as indicated by the accumulation of ^32^S^−^ and ^34^S^−^ secondary ion signal intensities. The close‐up image (small dashed square) shows an individual symbiosome. SM: symbiosome membrane; SS: symbiosome space; B: bacteroid; PC: plant cytosol. (b,c) The lower images show the corresponding TEM micrograph (b) and the overlay with the NanoSIMS image (c). The overlaid region is indicated in (a) by a white line. Scale bars, 1 μm. NanoSIMS: nanoscale secondary ion mass spectrometry; S: sulfur

**Figure 2:**
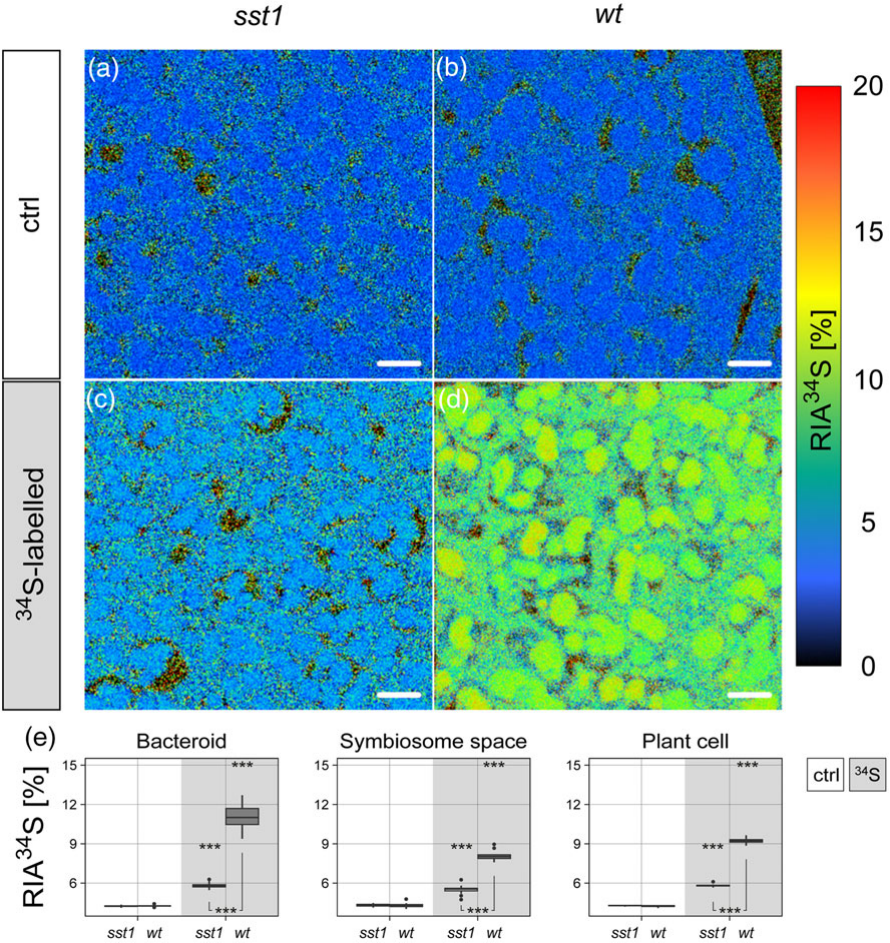
NanoSIMS high‐resolution analysis of ^34^S content, displayed as relative isotope abundance (RIA ^34^S = ^34^S/(^32^S + ^34^S), given in %) within nodule sections. Plants of the *sst1* (a,c) and wt (b,d) lines were supplied for 0 hr (crtl a,b) and 96 hr (c,d) with ^34^S‐enriched sulfate. Note that the speckled appearance of the isotopic composition within the SS, mimicking ^34^S enrichment for the natural abundance controls, is an artefact from inferior counting statistics on the per‐pixel basis. In the ROI‐analysis (see below), bias is overcome through accumulation of signal intensities from several pixels. (e) Results from ROI‐specific data evaluation of NanoSIMS images displayed in panels (a) to (d). Box‐plots summarize individual relative abundance values (min/max; first, second, and third quartiles) determined within bacteroids, SS, and plant cytosol. Asterisks indicate significant differences between control and ^34^S treatment of the same genotype (above) and between *sst1* and wt in the same treatment (below). *P* < 0.01 (**) and *P* < 0.001 (***) based on ANOVA (*n* = 8–149). Scale bars, 2 μm (a–d). NanoSIMS: nanoscale secondary ion mass spectrometry; S: sulfur; SS: symbiosome space; ROI: regions of interest

Image data were evaluated using the WinImage software package (v. 2.0.8) provided by Cameca. Prior to stack accumulation, the individual images were aligned to compensate for positional variations arising from primary ion beam and/or sample stage drift. Relative isotopic abundance values of ^34^S/(^32^S + ^34^S), given in % and designated as “RIA^34^S” throughout the text, were calculated from ^32^S and ^34^S signal intensities using the formula ^34^S/(^32^S + ^34^S) = ^34^S^‐^/(^32^S^−^ + ^34^S^−^). These calculations were performed on a per‐pixel basis for generation of the RIA^34^S distribution images (Figure 2a‐d) and on a per‐regions of interest (ROI) basis for numerical data evaluation (Figure 2e). For visualization of the relative elemental S content (Figure 1), ^32^S^−^ and ^34^S^−^ signal intensity distribution maps were accumulated. Owing to the smoothness of the analysed resin sections, the influence of sample topography on the S^−^ secondary ion signal intensities was considered to be negligible.

Individual ROI were manually defined based on the morphological features identifiable in the ^12^C^14^N^−^, ^31^P^−^, and (^32^S^−^ + ^34^S^−^) signal intensity distribution maps, as well as on the structural information gained from the TEM images. As outlined in the main text, ROIs were partitioned into bacteroids (B), SS, and plant cytosol (PC). For each of these subcategories between 8 and 149 individual ROIs were analysed per measurement (Table S4).

## 3 RESULTS AND DISCUSSION

### 3.1 Significant amounts of S are translocated from the host to the symbiont via SST1

**Figure 3:**
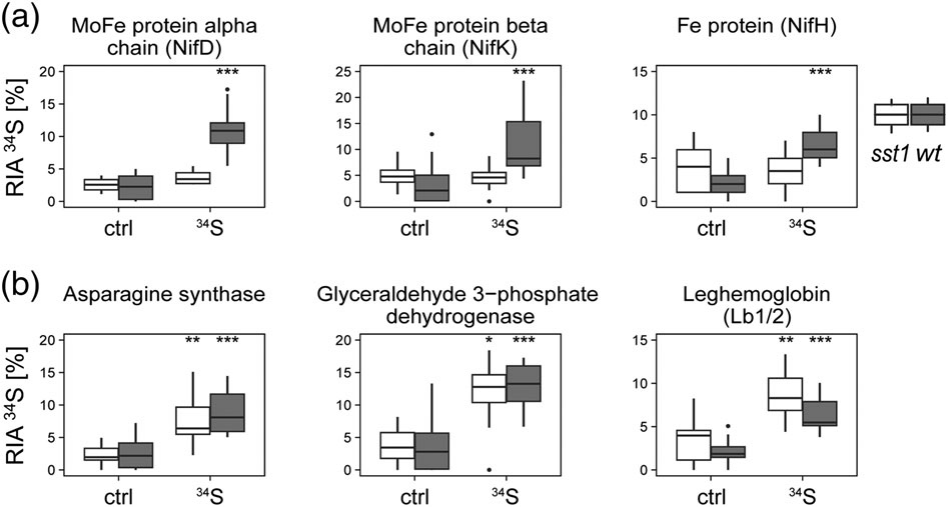
Relative isotope abundance of ^34^S (RIA ^34^S). (a) Different bacterial nitrogenase subunits. (b) Plant nodulins. RIA ^34^S = ^34^S/(^32^S + ^34^S). Asterisks indicate significant differences between natural abundance control (ctrl) and ^34^S‐sulfate treatment of the same genotype. *P* < 0.05 (*), *P* < 0.01 (**), and *P* < 0.001 (***), based on ANOVA (*n* = 3–4)

To visualize and quantify S transport in L. japonicus from the nodule PC to the bacteroids (Figure 2) and the subsequent incorporation of reduced S into the nitrogenase complex (Figure 3), we utilized sulfate enriched with the stable isotope ^34^S. The ^32^S isotope has the highest terrestrial abundance with 95.02%, followed by ^34^S (4.21%), ^33^S (0.75%), and ^36^S (0.02%; Tcherkez & Tea, 2013). Wild‐type (wt) plants were exposed for 0 hr (control) and 96 hr to ^34^S‐sulfate, and subsequently, nodules were analysed by NanoSIMS. This analysis demonstrated a 20‐fold increase of ^32^S^‐^ and ^34^S^‐^ secondary ion signal intensities in bacteroids compared with the nodule cytosol of the wt plants (Figure 1). Furthermore, increased levels of ^34^S in the plant cell cytosol, SS, and bacteroids after 96 hr were observed, being highest (~10% relative abundance) in the bacteroids (Figure 2d,e). This corroborates the important role of sulfate for the symbiont, consistent with previous data showing that a defective *sst1* gene causes early nodule senescence (Krusell et al., 2005). In addition, these data demonstrate that sulfate taken up by the plant is transported across the SM and accumulated at high levels in the bacteroids. To verify that sulfate transport across the SM in the *sst1‐1* mutants is impeded, we compared nodules of L. japonicus *sst1‐1* mutants incubated with ^34^S‐sulfate for 96 hr with nodules of wt plants (Figure 2c–e). We found that the abundance of ^34^S was significantly (*P* ≤ 0.001) reduced, reaching only about 6 at% (i.e., ~2% higher than natural abundance) in the bacteroids of the mutant nodules (Figure 2e), confirming the importance of sulfate transport for SNF. In addition, the *sst1‐1* nodules are not capable of efficient N_2_ fixation and displayed lower levels of the Fe‐protein of nitrogenase and of total S, as well as reduced growth under symbiotic conditions, as compared with the wt (Krusell et al., 2005). However, this mutation was reported not to affect growth of L. japonicus under nonsymbiotic conditions (Krusell et al., 2005).

**Figure 4:**
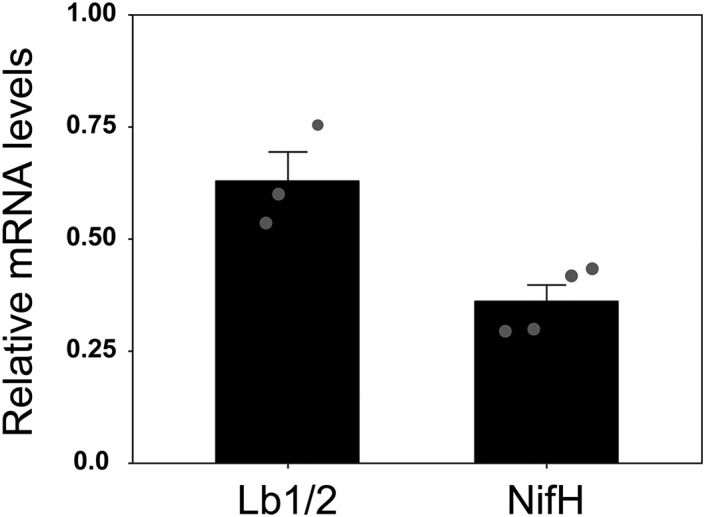
Expression of mRNA levels of leghemoglobins (Lb1/2) and nitrogenase Fe‐protein (NifH). Transcript levels in nodules of the *sst1* mutant are expressed relative to those of the wt plants (*R* = 1) using ubiquitin for Lotus japonicus and *sigA* for Mesorhizobium loti as reference genes. The dot plot overlay shows the distribution ofevery data point. Values are means ± SD (*n* = 3‐4)

A global reduction of S‐uptake into the *sst1‐1* mutant nodules, including the PC, was expected because the plant is suffering from impaired nodule function, causing a general shutdown of important symbiotic processes (Krusell et al., 2005; Pladys & Vance, 1993). Hence, the plant's sulfate partitioning seems to be reprogrammed, which leads to a global reduction of S levels, including cytoplasmic vacuolation, lysis of bacteroids (Krusell et al., 2005; Figure S1), reduced synthesis of nodulins such as Lbs (Krusell et al., 2005; Figure S2), and ultimately nodule senescence (Pladys & Vance, 1993). Impaired nodule functioning is also supported by the decrease in the contents of nitrogenase subunits (Figure S3a), as well as in the mRNAs encoding Lb1/2 and the Fe‐protein of nitrogenase (Figure 4). Noteworthy, the higher the number of S‐containing amino acids in the nitrogenase subunits, the lower the relative protein abundance in bacteroids of the *sst1* mutant compared with the wt (Figure S3b). This indicates a decrease in the *de novo* biosynthesis of nitrogenase and Lb as a direct result of S deprivation.

**Figure 5:**
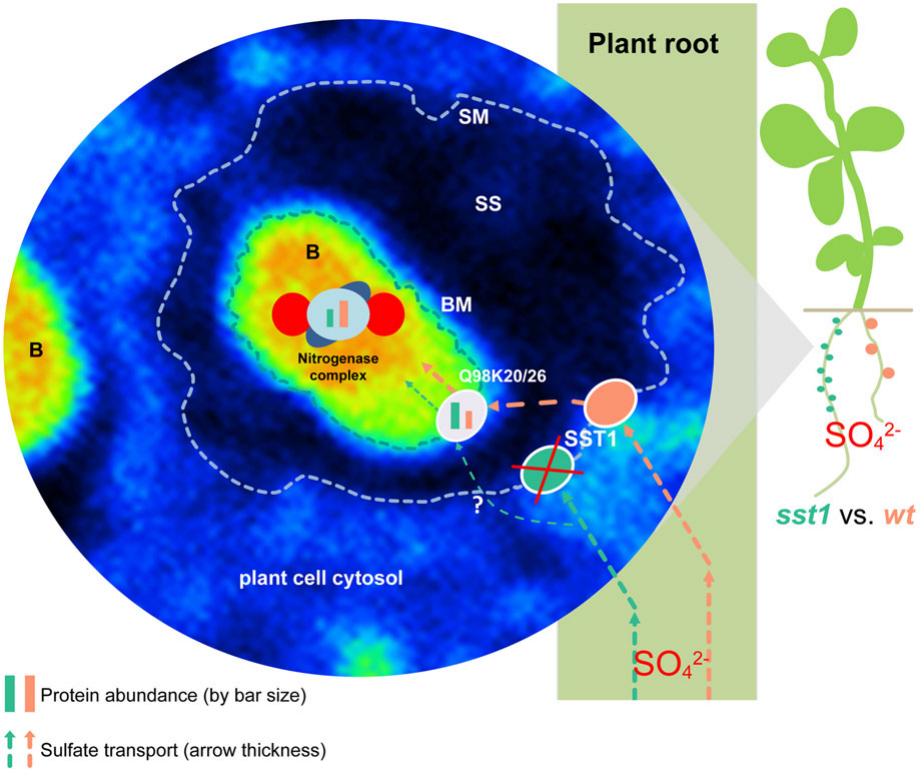
Schematic overview of the major results of NanoSIMS and SILIP‐MS data, depicting how S deficiency, brought about by the lack of SST1, leads to inhibition of nitrogenase biosynthesis. SM: symbiosome membrane; SS: symbiosome space; B: bacteroid; BM: bacteroid membrane; ?: uncontrolled diffusion. UniProt accessions Q98K20 (mlr1672) and Q98K26 (mlr1666), bacteroid sulfate transporting ATPases. NanoSIMS: nanoscale secondary ion mass spectrometry; S: sulfur

Interestingly, we found that two rhizobial proteins with sulfate‐transporting ATPase activity, Q98K26 (mlr1666) and Q98K20 (mlr1672), show slightly and significantly enhanced levels, respectively, in the mutant and may possibly be involved in the regulation of sulfate uptake (Figure S4). Despite the expected markedly lower relative ^34^S abundance in the bacteroids of the *sst1‐1* mutant, there was still a significant increase compared with the control (Figure 2e). This might be explained by uncontrolled sulfate diffusion through the SM (illustrated in Figure 5), indicating that active transport is necessary in order to reach increased S levels such as in the wt.

The much greater accumulation of S in the bacteroids of wt nodules compared with the plant tissue was unexpected although S‐metabolism in nodules is very active and complex (Becana, Wienkoop, & Matamoros, 2018). Thus, the thiol tripeptide glutathione is very abundant in the bacteroids and is essential for SNF (El Msehli et al., 2011; Matamoros et al., 2003; Muglia, Comai, Spegazzini, Riccillo, & Aguilar, 2008). However, it is unclear whether glutathione can be transported through the SM in addition to being synthesized in the bacteroids by the sequential action of γ‐glutamylcysteine synthetase and glutathione synthetase. Moreover, it was demonstrated that N_2_‐fixing nodules are the main site of Cys accumulation in L. japonicus (Kalloniati et al., 2015; Matamoros, Moran, Iturbe‐Ormaetxe, Rubio, & Becana, 1999). Taking this finding together with our results, we suggest that the bacteroids are the main site of Cys accumulation and synthesis in active nodules, as the large amount of sulfate transported to the bacteroids needs to be converted into amino acids for protein biosynthesis. Hence, the data indicate that the plant trades with the bacteroids sugars and sulfate in exchange for nitrogen. We show that bacteroids have a high demand for S that relies on active transport through SST1, an indication that sulfate is essential for regulation and functioning of SNF.

### 3.2 Sulfur taken up by the plant is used for nitrogenase biosynthesis

To verify that SNF and, more specifically, the *de novo* synthesis of nitrogenase was directly linked to sulfate availability, we monitored ^34^S‐incorporation via Met or Cys into nitrogenase proteins using MS. Analysis of the bacteroid proteins of wt nodules revealed that, similar to the findings from our NanoSIMS measurements, the relative ^34^S‐isotope abundance of nitrogenase subunits was increased up to 10% (Figure 3a). These data provided, therefore, additional evidence for the transport of sulfate across the SM, as well as for its utilization for protein biosynthesis in the bacteroids. This was further confirmed by our results with the mutant, which showed that the relative abundance of ^34^S was not elevated and hence that the *de novo* synthesis and incorporation into nitrogenase was hampered (Figure 3a).

To further examine this hypothesis, we analysed whether, and to what extent, plant nodulin biosynthesis (independent of SST1) was also restricted in the *sst1‐1*‐deficient mutant. For this purpose, we determined the ^34^S‐incorporation into Lb1/2, asparagine synthase, and nodule‐enhanced glyceraldehyde‐3‐phosphate dehydrogenase. The relative abundance of ^34^S significantly increased in these enzymes, in both the wt and mutant nodules of labelled plants (Figure 3b), indicating that *de novo* synthesis of plant nodulins, unlike that of the bacteroid nitrogenase subunits, was not affected by S limitation. Hence, the lack of detectable nitrogenase *de novo* synthesis together with the very low levels of ^34^S RIA in the bacteroids of the *sst1* mutants suggest that a high sulfate abundance through active transport is required in order for the bacteroids to synthesize nitrogenase.

Anderson and Spencer (1950) showed that sulfate deficiency drastically reduced SNF in clover. The deficiency led to a lower total protein content and fewer nodules in comparison with plants provided with a sulfate source. The same authors found that the addition of nitrate to sulfate‐deficient clover did not improve plant growth, indicating that the restricted growth of sulfate‐deficient clover was not due to poor N_2_ fixation but caused by the lack of sulfate in the host legume. Andrew (1977) analysed the effect of sulfate shortage on growth and N concentrations in tropical and temperate legumes, reaching similar conclusions. They showed that N concentrations increased after sulfate supply. Likewise, Zhao et al. (1999) found that SNF was very sensitive to sulfate deficiency because the addition of sulfate to pea plants growing on sulfate‐deficient soil doubled the amount of fixed N at all growth stages of the plant. Whether this was due to a direct effect on SNF or to an effect on the host plants remains unclear. More recently, it has been shown that S deficiency in Trifolium repens reduced SNF due to a lower nodule development and as a result of low nitrogenase and Lb production (Varin, Cliquet, Personeni, Avice, & Lemauviel‐Lavenant, 2010). Our data thus suggest that sulfate deficiency has a direct impact on SNF and limits protein biosynthesis without detrimental effects to the plant.

Taken together, these findings demonstrate that S deficiency in the bacteroids caused by the lack of SST1 leads to a direct effect on SNF by the inhibition of nitrogenase synthesis as summarized in Figure 5. Thus, both the host plant and SNF will be strongly affected by sulfate deficiency in the bacteroids, which would naturally be caused by a general sulfate deficiency in the soil. In other words, this agronomically relevant symbiosis is able to compensate for N‐limitation but not for S‐limitation. Functioning of this symbiosis is clearly hampered under sulfate deficiency, a frequently overlooked issue for which we provide here an explanation by directly linking sulfate incorporation to nitrogenase biosynthesis.

## AUTHOR CONTRIBUTIONS

S. S. made the protein and MS analyses, assessed the data, and prepared the samples for TEM and NanoSIMS analyses; A. S. carried out the NanoSIMS analysis, assessed the data, and revised the manuscript; M. B. made the transcript analysis and revised the manuscript; M. W. contributed to NanoSIMS method development and to the manuscript revision; D. W. contributed to the conception of NanoSIMS analysis and to the manuscript revision; S. W. conceived and designed the work and contributed to MS data assessment; S. S., M. B., and S. W. wrote the manuscript.

## Supporting information


**Figure S1.** Light micrographs of a nodule of L. japonicus. Nodule semi‐thin sections (1 μm) were collected on glass slides and stained with toluidine blue. (a) wt plants. (b) *sst1* mutant showing lower density of infected cells (blue) and signs of elevated cytoplasmic vacuolation and lysis of bacteroids. Scale bars, 75 μm.
**Figure S2.** Relative quantification of the three Lb isoforms and comparison of their abundances in nodules of *sst1* and wt plants. LFQ, label‐free quantification (MaxQuant) as described in Methods. Values (see also Table S1b) are means ± SE (*n* = 16–17). *P* < 0.01 (**), *P* < 0.001 (***) based on ANOVA.
**Figure S3.** Relative quantification of the three nitrogenase subunits and their S‐containing peptides. (a) Comparison of the abundance of nitrogenase subunits in nodules of *sst1* mutant and wt plants. (b) Correlation between the total number of S‐containing amino acids of the three nitrogenase subunits (black columns) and the average percent abundance (measured intensities) of peptides with these S‐containing amino acids (grey squares) in *sst1* mutants compared to wt plants [*sst1*/wt, taken from (a)]. LFQ, label‐free quantification (MaxQuant) as described in Methods. Nitrogenase sequence information was taken from UniProt: Q98AP5 or NifK (β subunit of MoFe‐protein); Q98AP6 (α subunit of MoFe‐protein or NifD); and Q98AP7 (subunit of Fe‐protein or NifH). Values are means ± SE (*n* > 20). *P* < 0.001 (***) based on ANOVA.
**Figure S4.** Relative protein abundances in the *sst1* and wt plants of the bacteroid sulfate‐transporting ATPases, Q98K26 (mlr1666) and Q98K20 (mlr1672). LFQ, label‐free quantification. *P* < 0.05 (*) based on ANOVA (*n* > 20).

**Table S1a.** MaxQuant information on protein and peptide identification used in Figure 3 and Figures S2 and S3.
**Table S1b.** MaxQuant information of protein Label‐Free Quantification (LFQ intensities) used in Figures S2 and S3. rep, biological replicate; ND, not determined.
**Table S2a.** Data matrix of RIA value calculations, extracted from the MS analyses of the bacteroid protein fraction at time point (TP) 0 hr and TP 96 hr of ^34^S‐labelled nodules shown in Figure 3a.
**Table S2b.** Data matrix of RIA value calculations, extracted from the MS analyses of the plant protein fraction at time points 0 hr and 96 hr of ^34^S‐labelled nodules shown in Figure 3b.
**Table S3.** Primers used for quantitative reverse‐transcription PCR.
**Table S4a.** Data matrix of ROI of NanoSIMS analysis of wt nodules, control plants.
**Table S4b.** Data matrix of ROI of NanoSIMS analysis of wt nodules, 96 hr ^34^S‐labelled plants.
**Table S4c.** Data matrix of ROI of NanoSIMS analysis of *sst1* nodules, control plants.
**Table S4d.** Data matrix of ROI of NanoSIMS analysis of *sst1* nodules, 96 hr ^34^S‐labelled plants.
