# Supplementary material for: Sulfate is transported at significant rates through the symbiosome membrane and is crucial for nitrogenase biosynthesis
Source: Plant Cell Environ. 2019 Jan 28;42(4):1180–9. doi: 10.1111/pce.13481 (PMC6446814; doi:10.1111/pce.13481)
Supplement: Supplementary file 1 — Figure S1. Light micrographs of a nodule of L. japonicus. Nodule semi‐thin sections (1 μm) were collected on glass slides and stained with toluidine blue. (a) wt plants. (b) sst1 mutant showing lower density of infected cells (blue) and signs of elevated cytoplasmic vacuolation and lysis of bacteroids. Scale bars, 75 μm. Figure S2. Relative quantification of the three Lb isoforms and comparison of their abundances in nodules of sst1 and wt plants. LFQ, label‐free quantification (MaxQuant) as described in Methods. Values (see also Table S1b) are means ± SE (n = 16–17). P < 0.01 (**), P < 0.001 (***) based on ANOVA. Figure S3. Relative quantification of the three nitrogenase subunits and their S‐containing peptides. (a) Comparison of the abundance of nitrogenase subunits in nodules of sst1 mutant and wt plants. (b) Correlation between the total number of S‐containing amino acids of the three nitrogenase subunits (black columns) and the average percent abundance (measured intensities) of peptides with these S‐containing amino acids (grey squares) in sst1 mutants compared to wt plants [sst1/wt, taken from (a)]. LFQ, label‐free quantification (MaxQuant) as described in Methods. Nitrogenase sequence information was taken from UniProt: Q98AP5 or NifK (β subunit of MoFe‐protein); Q98AP6 (α subunit of MoFe‐protein or NifD); and Q98AP7 (subunit of Fe‐protein or NifH). Values are means ± SE (n > 20). P < 0.001 (***) based on ANOVA. Figure S4. Relative protein abundances in the sst1 and wt plants of the bacteroid sulfate‐transporting ATPases, Q98K26 (mlr1666) and Q98K20 (mlr1672). LFQ, label‐free quantification. P < 0.05 (*) based on ANOVA (n > 20). [file PCE-42-1180-s001.pdf]

Figure S1

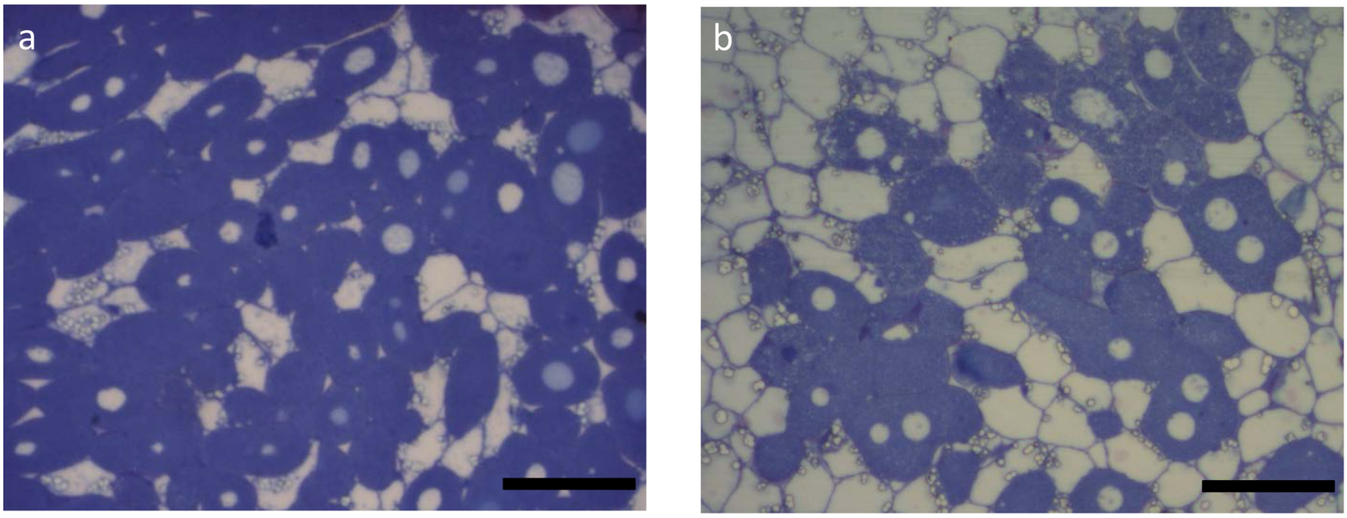

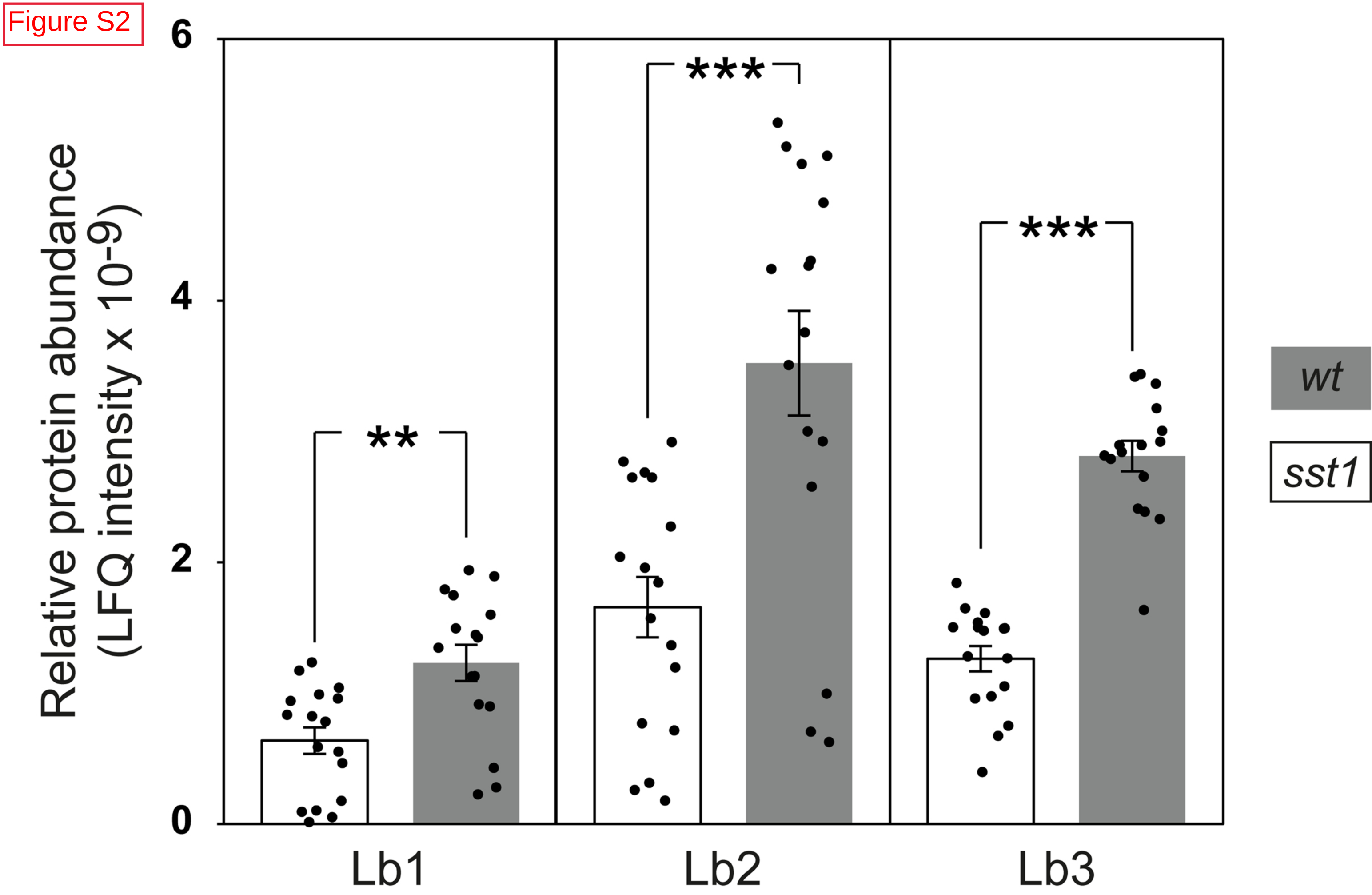
Figure S2
Relative protein abundance
(LFQ intensity x 10-9)
0
2
4
6
**
***
***
Lb1
Lb2
Lb3
wt
sst1

Figure S3

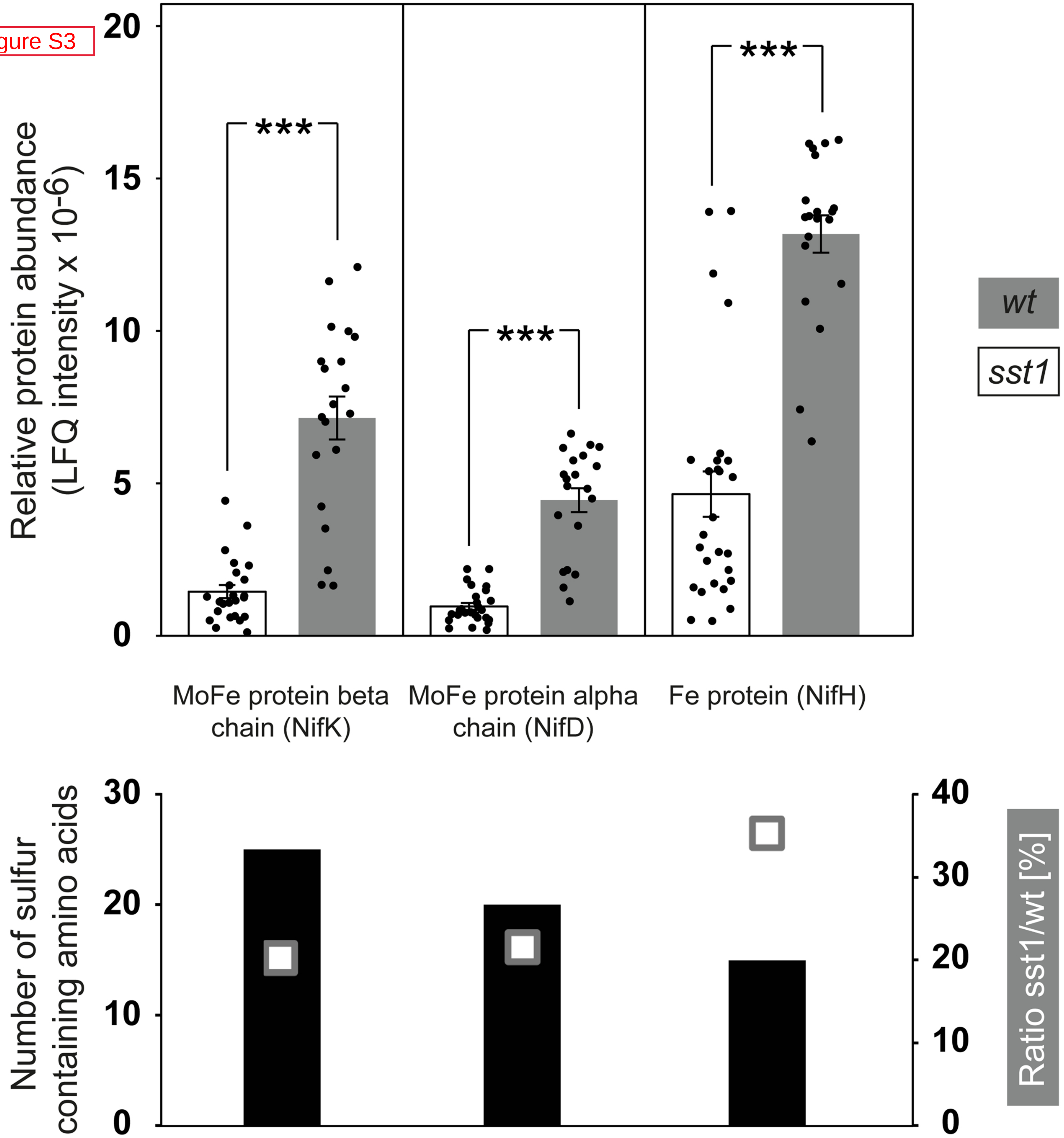

Relative protein abundance
(LFQ intensity x 10-6)
20
15
10
5
0
***
***
***
wt
sst1
MoFe protein beta chain (NifK)
MoFe protein alpha chain (NifD)
Fe protein (NifH)
Number of sulfur containing amino acids
30
20
10
0
40
30
20
10
0
Ratio sst1/wt [%]

Figure S4

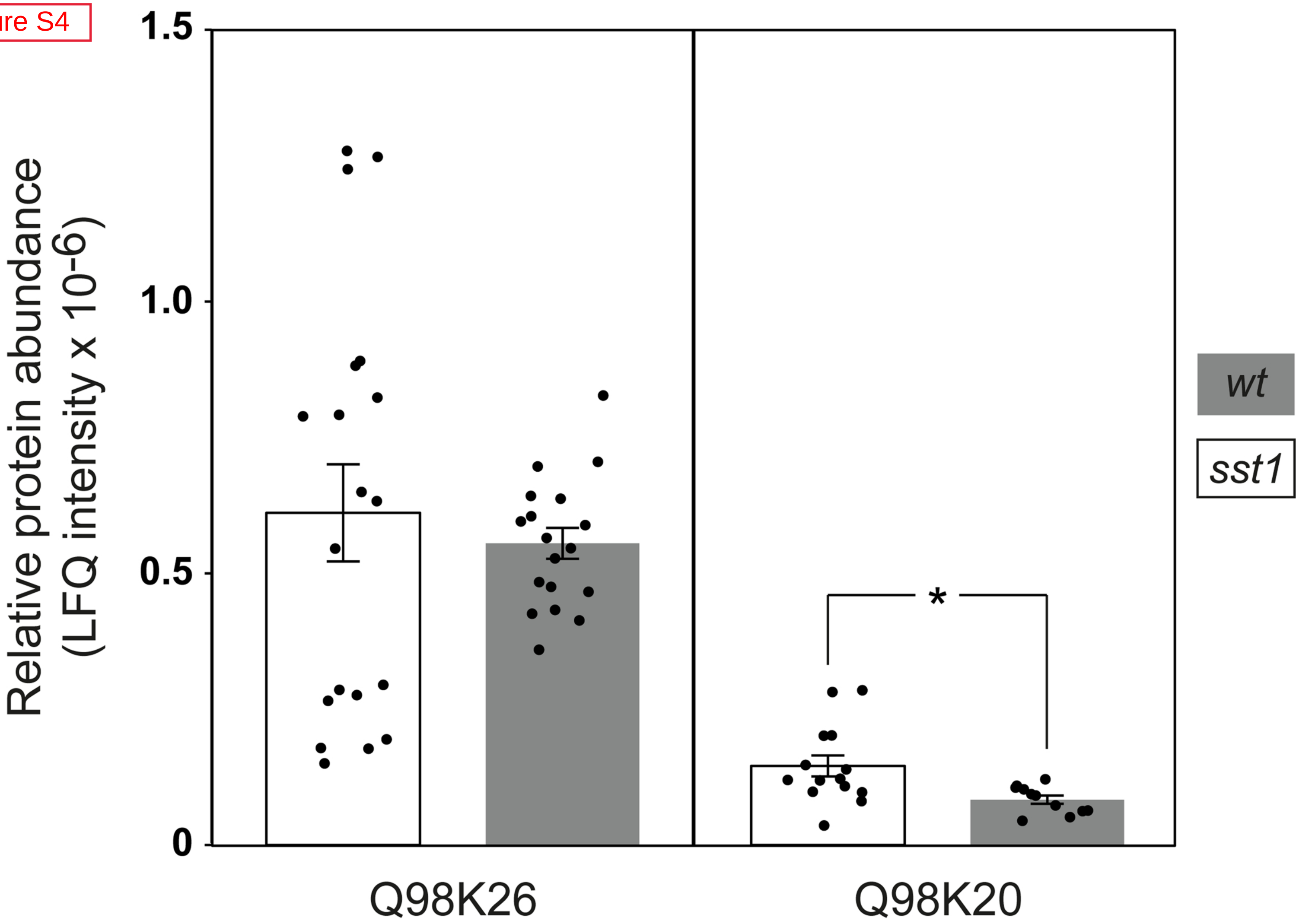
1.5
1.0
0.5
0
Relative protein abundance
(LFQ intensity x 10-6)
*
Q98K26
Q98K20
wt
sst1
