# Supplementary material for: Sulfate is transported at significant rates through the symbiosome membrane and is crucial for nitrogenase biosynthesis
Source: Plant Cell Environ. 2019 Jan 28;42(4):1180–9. doi: 10.1111/pce.13481 (PMC6446814; doi:10.1111/pce.13481)
Supplement: Supplementary file 2 — Table S1a. MaxQuant information on protein and peptide identification used in Figure 3 and Figures S2 and S3. Table S1b. MaxQuant information of protein Label‐Free Quantification (LFQ intensities) used in Figures S2 and S3. rep, biological replicate; ND, not determined. Table S2a. Data matrix of RIA value calculations, extracted from the MS analyses of the bacteroid protein fraction at time point (TP) 0 hr and TP 96 hr of 34S‐labelled nodules shown in Figure 3a. Table S2b. Data matrix of RIA value calculations, extracted from the MS analyses of the plant protein fraction at time points 0 hr and 96 hr of 34S‐labelled nodules shown in Figure 3b. Table S3. Primers used for quantitative reverse‐transcription PCR. Table S4a. Data matrix of ROI of NanoSIMS analysis of wt nodules, control plants. Table S4b. Data matrix of ROI of NanoSIMS analysis of wt nodules, 96 hr 34S‐labelled plants. Table S4c. Data matrix of ROI of NanoSIMS analysis of sst1 nodules, control plants. Table S4d. Data matrix of ROI of NanoSIMS analysis of sst1 nodules, 96 hr 34S‐labelled plants. [file PCE-42-1180-s002.zip › Table S3.docx]

**Table S4.** Primers used for quantitative reverse-transcription PCR.

*LjLb1/2* (fw)^*^ 5`-TTTGAGCACTGCTTGGGGAGTAGCT-3`

*LjLb1/2* (rev)^*^ 5`-CATTGCCTTCTTAATTGCAG-3`

*LjUBQ* (fw) 5`-TTCACCTTGTGCTCCGTCTTC-3`

*LjUBQ* (rev) 5`-AACAACAGCACACACAGACAA-3`

*LjATPsyn* (fw) 5`-AACACCACTCTCGATCATTTCTCTG-3`

*LjATPsyn* (rev) 5`-CAATGTCGCCAAGGCCCATGGTG-3`

*nifH* (fw) 5`-TCCAAGCTCATCCACTTCGTG-3`

*nifH* (rev) 5`-AGTCCGGCGCATACTGGATTA-3`

*sigA* (fw) 5`-GCCCTCTGCTCGACCTTTCC-3`

*sigA* (rev) 5`-AGCATCGCCATCGTGTCCTC-3`

^*^Primer sequences according to Ott et al. (2015). They have been published as specific for LjLb1, but are recognizing both LjLb1 and LjLb2.
